# Ethnic differences in the association of fat and lean mass with bone mineral density in the Singapore population

**DOI:** 10.1186/1753-6561-6-S4-P43

**Published:** 2012-07-09

**Authors:** AE Teo, AC Ng, K Venkataraman, ES Tai, YS Lee, EY Khoo, CM Khoo, SA Sadananthan, SS Velan, V Zagorodnov, YS Chong, P Gluckman, MK Leow

**Affiliations:** 1 Agency for Science, Technology and Research, Singapore; 2 University of Cambridge, UK; 3 Department of Endocrinology, Singapore General Hospital, Singapore; 4 Department of Obstetrics and Gynaecology, Yong Loo Lin School of Medicine, National University of Singapore, Singapore; 5 Department of Medicine, Yong Loo Lin School of Medicine, National University of Singapore, Singapore; 6 Singapore Institute for Clinical Sciences, Agency for Science, Technology and Research, Singapore; 7 Department of Paediatrics, Yong Loo Lin School of Medicine, National University of Singapore, Singapore; 8 Singapore Bioimaging Consortium, Agency for Science, Technology and Research, Singapore; 9 Department of Computer Engineering, Nanyang Technological University, Singapore; 10 Department of Endocrinology, Tan Tock Seng Hospital, Singapore

## Introduction

Obesity and osteoporosis are two global health problems with pronounced morbidity and mortality. While body weight appears to mitigate the development of osteoporosis, whether excess body fat promotes or protects against osteoporosis remains a conundrum. The effect of ethnicity on these associations has also been understudied. We hypothesize that (1) fat mass (FM) and lean mass (LM) are independently associated with bone mineral density (BMD) and that (2) ethnic differences exist in the association of FM and LM with BMD among Chinese, Malay and Indian subjects.

## Methods

We evaluated 150 overweight male subjects aged ≥21 years with body mass index ≥25 from 3 ethnic groups (Chinese =73; Malays =41; Indians =36). BMD in five regions (lumbar spine, femoral neck, total hip, ultra-distal radius and one-third radius), FM and LM were measured by dual-energy X-ray absorptiometry (DEXA) using a Hologic Discovery Wi densitometer. Whole abdomen subcutaneous and visceral fat volumes were determined by magnetic resonance imaging (MRI) and a validated segmentation algorithm. Linear regression models were developed to test the association of FM/LM with BMD, and univariate ANOVA was used to test for interaction between ethnicity and FM/LM with BMD.

## Results

After adjusting for age and height, LM was positively correlated with BMD in all three ethnicities, but in different skeletal sites: weight-bearing regions (femoral neck, hip) in Chinese, and non-weight-bearing regions (ultra-distal and one-third radius) in Malays and Indians. A negative correlation between FM and BMD was observed consistently in all regions for the Indians, especially at the hip. Visceral fat was negatively correlated with BMD, being most pronounced among Chinese and least for Malays. The interaction models revealed that with each unit of LM, Malays showed a greater increment in BMD than Chinese and Indian subjects at the ultra-distal radius.

## Conclusions

Our findings suggest that FM and LM affect BMD in opposite directions, with different physiological reasons modulating this relationship. Substantial ethnic differences were observed in the association of FM and LM with BMD. These results may help explain the variation in hip and wrist fracture rates by ethnicity, and may warrant ethnic-specific clinical recommendations.

